# A Comparison of Lysosomal Enzymes Expression Levels in Peripheral Blood of Mild- and Severe-Alzheimer’s Disease and MCI Patients: Implications for Regenerative Medicine Approaches

**DOI:** 10.3390/ijms18081806

**Published:** 2017-08-19

**Authors:** Francesco Morena, Chiara Argentati, Rosa Trotta, Lucia Crispoltoni, Anna Stabile, Alessandra Pistilli, Angela di Baldassarre, Riccardo Calafiore, Pia Montanucci, Giuseppe Basta, Anna Pedrinolla, Nicola Smania, Massimo Venturelli, Federico Schena, Fabio Naro, Carla Emiliani, Mario Rende, Sabata Martino

**Affiliations:** 1 Department of Chemistry, Biology and Biotechnology, Biochemistry and Molecular Biology Unit, University of Perugia, Perugia 06123, Italy; effemorena@gmail.com (F.M.); chiara.argentati89@gmail.com (C.A.); rosa.trotta@tiscali.it (R.T.); carla.emiliani@unipg.it (C.E.); 2 Department of Surgery and Biomedical Sciences, Section of Human, Clinical and Forensic Anatomy, School of Medicine, University of Perugia, Perugia 06132, Italy; lucia.crispoltoni@gmail.com (L.C.); anna.stabile@unipg.it (A.S.); alessandra.pistilli@unipg.it (A.P.); mario.rende@unipg.it (M.R.); 3 Department of Aging Medical Science, University of G. d’Annunzio, Chieti e Pescara, Chieti 66100, Italy; a.dibaldassarre@unich.it; 4 Department of Medicine, Section of Cardiovascular, Endocrine and Metabolic Clinical Physiology and Laboratory for Endocrine Cell Transplants and Bio-hybrid Organs, University of Perugia, Perugia 06132, Italy; riccardo.calafiore@unipg.it (R.C.); piamontanucci@hotmail.com (P.M.); gius.basta@gmail.com (G.B.); 5 Department of Neurosciences, Biomedicine and Movement Sciences, University of Verona, Verona 37134, Italy; anna.pedrinolla@univr.it (A.P.); nicola.smania@univr.it (N.S.); massimo.venturelli@univr.it (M.V.); federico.schena@univr.it (F.S.); 6 Department of Anatomical, Histological, Forensic and Orthopedic Sciences, Sapienza University of Roma, Roma 06100, Italy; fabio.naro@uniroma1.it

**Keywords:** β-Hexosaminidase, β-Galactosidase, β-Galactosylcebrosidase, β-Glucuronidase, Cathepsin S, Cathepsin D, Cathepsin B, Cathepsin L, dementia, neurodegeneration, aging

## Abstract

The association of lysosomal dysfunction and neurodegeneration has been documented in several neurodegenerative diseases, including Alzheimer’s Disease (AD). Herein, we investigate the association of lysosomal enzymes with AD at different stages of progression of the disease (mild and severe) or with mild cognitive impairment (MCI). We conducted a screening of two classes of lysosomal enzymes: glycohydrolases (β-Hexosaminidase, β-Galctosidase, β-Galactosylcerebrosidase, β-Glucuronidase) and proteases (Cathepsins S, D, B, L) in peripheral blood samples (blood plasma and PBMCs) from mild AD, severe AD, MCI and healthy control subjects. We confirmed the lysosomal dysfunction in severe AD patients and added new findings enhancing the association of abnormal levels of specific lysosomal enzymes with the mild AD or severe AD, and highlighting the difference of AD from MCI. Herein, we showed for the first time the specific alteration of β-Galctosidase (Gal), β-Galactosylcerebrosidase (GALC) in MCI patients. It is notable that in above peripheral biological samples the lysosomes are more sensitive to AD cellular metabolic alteration when compared to levels of Aβ-peptide or Tau proteins, similar in both AD groups analyzed. Collectively, our findings support the role of lysosomal enzymes as potential peripheral molecules that vary with the progression of AD, and make them useful for monitoring regenerative medicine approaches for AD.

## 1. Introduction

Alzheimer’s disease (AD) is the most common form of dementia in elderly individuals and, at the present, it is one of the major challenges of the global public health [1]. However, the pathogenic complexity of the disease together with some clinical signs, common to other neurodegenerative diseases (e.g., elderly dementia, fronto-temporal dementia) [1,2,3,4,5,6], make the diagnosis and the development of effective treatments for AD difficult.

In the last decade, effort has been made to explore innovative regenerative medicine approaches for AD. This includes: replacement cell therapy approaches by implantation of different types of stem cells in AD animal models [7]; production of neuronal devise with innovative nanomaterials for improving or delay neurologic impairment [8]; new drug delivery approaches such as the use of an efficient monoclonal antibody against the amyloid-β peptide [9]. In the meantime, new potential diagnostic tools are also being explored [10,11,12]. These include: (i) the development of innovative high sensitive technologies to reveal early neurological alteration [13,14]; and (ii) the development of non-invasive biological assays allowing the identification of subjects at early stages of AD or the recognition of AD from others dementia (e.g., mild cognitive impairment: MCI). Therefore, effort has been made to identify new molecules that strictly vary with the AD progression and are easily monitored also in non-neurological samples. Thus, the screening of cytokines, stress oxidative molecules, and microRNAs are under evaluation as potential peripheral biomarkers of AD [15,16,17].

In this issue, altered levels of lysosomal enzymes in AD patients have been attracted the interest of many research groups [18,19,20,21,22,23,24,25,26,27,28]. Abnormal lysosomes function in AD patients has been observed both in the specific lysosomal enzymes expression [21,22,23,24,25,26,27] and in the autophagy machinery dysfunction [18,19,20,28,29]. For instance, a great accumulation of lysosome-like organelles was described within swollen axons close to region in contact with the amyloid plaques, demonstrating that they were lacking of several proteases and therefore un-effective in proteins degradation, including amyloid storages [21]. Other authors have demonstrated the presence of some proteases (e.g., Cathepsin B, Cathepsin S, and Cathepsin D) in the deposition of amyloid plaques [24,25].

For several years we are studying the association of expression of some lysosomal enzymes (β-Hexosaminidase, and β-Galactosidase, α-mannosidase, Cathepsin D) and severe AD [20,26,27]. In particular we have demonstrated an up-regulation of lysosomal glycohydrolases α-mannosidase, β-Hexosaminidase, and β-Galactosidase in skin fibroblasts from sporadic, familial AD patients and presymptomatic subjects [30]. Altered levels of β-Galactosidase and β-Hexosaminidase in AD and type-2 diabetes mellitus (T2D) patients compared to T2D or control subjects were also observed. In these patients, the ROC (Receiver Operating Characteristic) curves statistical analyses, allows to use both enzymes to discriminate AD patients from controls and AD-T2D from T2D patients [15,26,27].

Here, we explored whether the expression levels of lysosomal enzymes could be associated with AD at different stages of disease progression (mild and severe) or with mild cognitive impairment (MCI) patients and, in turn, if it could have diagnostic potential.

We conducted a screening of a collection of two classes of lysosomal enzymes selected on the basis of our previous studies: the glycohydrolases (β-Hexosaminidase, β-Galactosidase, β-Galactosylcebrosidase, β-Glucuronidase) and proteases (Cathepsin S, Cathepsin D, Cathepsin B, and Cathepsin L) [26,30,31,32,33]. The analyses were performed in a blood sample consisting of a small sample of peripheral blood mononuclear cells (PBMCs) and blood plasma from a cohort of: (i) AD patients at different stages of the disease (mild AD and severe AD); (ii) mild cognitive impairment (MCI) patients; and (iii) healthy age-matched control group of subjects without cognitive impairment.

We documented for the first time an overall alteration of the aforementioned lysosomal enzymes in AD and MCI patients. We confirmed altered levels of the lysosomal enzymes in severe AD patients. Moreover, new findings showed for the first time a sensitive association of the above-mentioned proteins expression with the mild AD and MCI patients. Collectively, our results support the inclusion of lysosomal enzymes as potentials peripheral molecules that vary with the stages of progression of AD.

## 2. Results

### 2.1. Assessment of Lysosomal Enzymes in Peripheral Blood from Mild and Severe AD Patients and MCI Patients: Experimental Platform

#### 2.1.1. Patients Enrolled in the Study

This study involved a total of 59 subjects distributed in the following groups: MCI (*n* = 10), mild AD (*n* = 19), severe AD (*n* = 17) and age-matched controls (*n* = 13). The four groups were from the same geographic area and homogeneous for age, except for the severe AD patients who were significantly older than the control (CTR) (** *p* < 0.001) (CTR = 76 ± 4, MCI = 73.8 ± 2.9, mild AD = 77.6 ± 6.5 and severe AD = 85.62 ± 6.2 years old), gender (M/F; CTR = 5/8, MCI = 6/4, mild AD = 7/12, severe AD = 10/7). The CDR and MMSE scores evaluated the cognitive impairment in MCI and AD subjects. MCI patients have a CDR = 0 and MMSE = 24.8 ± 1.15; mild AD patients have a CDR = 1–2 and MMSE = 19.1 ± 3; severe AD patients have CDR = 3 and MMSE = 16.29 ± 5.18. The neuropsychological- and cognitive-profiling tests revealed that patients with MCI were characterized by impairments in one or more cognitive domains. These patients exhibited no clear signs of dementia, a preserved independence in the execution of the functional activities of daily living (~80%) and only few neuropsychiatric symptoms, such as depression and anxiety (NPI = 11). Patients with mild AD exhibited similar neuropsychiatric symptoms of the MCI patients, but worse executive functions and a reduced independence. The instrumental activity of daily life (IADL) score was particularly reduced in the administration of own medications and a reduced ability to handle moneys. Patients in the severe AD group, presented extensive and severe neuropsychiatric disorders such as delusions, agitation, anxiety, irritability, disinhibition, and apathy. Moreover, these patients were severely impaired in the execution of the IADL, leading to a full dependence on the food preparation, housekeeping, and the utilization of public transportation. Moreover, all groups were matched for coexisting chronic conditions. Drugs for AD and other medications taken by the groups of MCI and AD patients are displayed in Table 1. Systolic blood pressure, glucose, and LDL were higher than the normal values.

The control group was composed of subjects diagnosed with no cognitive impairment based on MMSE scores (MMSE = 28.5 ± 2).

#### 2.1.2. Lysosomal Enzymes Screening

All subjects enrolled in the study were screened for peripheral blood levels of two classes of lysosomal enzymes: glycohydrolases (which includes: β-Hexosaminidase (Hex), β-Galctosidase (Gal), β-Galactosylcerebrosidase (GALC), β-Glucuronidase (Gluc)) and proteases (which includes Cathepsins S, D, B, L (CatS, CatD, CatB, CatL, respectively)). All lysosomal enzymes were selected based on our studies describing their association either with neurological diseases [4,32], with immune cell functions [33,34] or with AD pathophysiology [23,26,28,29].

We took advantage of a combination of simple and highly-sensitive methods, allowing measurements of lysosomal protein expression even in small biological samples [35,36,37,38,39,40,41,42,43,44,45], such as the blood plasma and PBMCs from patients. The latter represents a suitable tool for systematic analyses due to the non-invasive method of samples collection from patients and the easy samples processing in standard operating procedures.

The blood sample from each patient and control was handled to separate plasma and PBMCs that were then processed as described above. Both plasma and PBMCs were evaluated for the activity of glycohydrolases by fluorogenic assays, and for the expression of proteases by Western blotting and ELISA.

All measurements were performed using standard operating procedures developed in our laboratory that assure lysosomal enzymes activity assays with an intra- and inter-assay coefficients of variation in the 2% and 3.9% range, respectively [26,32].

Of note, the same samples from each patient were used to evaluate the expression of β-amyloid peptide and Tau proteins by Western blotting and ELISA.

### 2.2. β-Amyloid and Tau Proteins Expression in PBMCs from Mild- and Severe AD and MCI Patients

**Figure 1:**
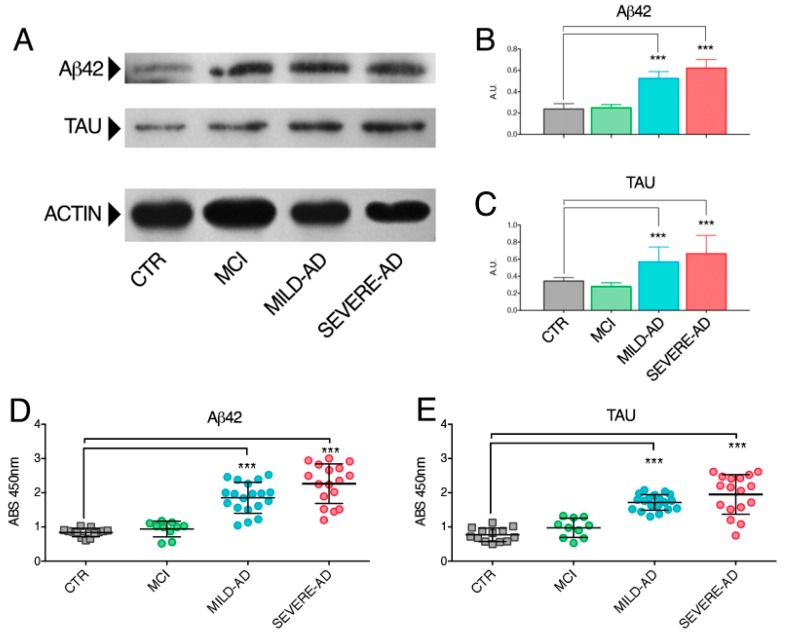
PBMCs from AD patients have primary hallmark of the disease: (**A**) Representative Western blotting of β-amyloid and Tau proteins analysis in PBMCs of mild AD, severe AD, MCI, and control subjects; (**B**,**C**) Densitometry analyses of both proteins were performed by the Fiji software (see Methods for details) and are reported as ratio toward β-actin protein, selected as reference. Each histogram is the mean ± SEM (standard error of the mean) of all samples analyzed in each group: CTR (*n* = 13); MCI (*n* = 10); mild AD (*n* = 19); and severe AD (*n* = 17); (**D**,**E**) Levels of β-amyloid and Tau proteins were determined in all samples of control group, MCI, mild AD and severe AD patients by using the ELISA assay. Results were expressed as the mean ± SEM of three independent experiments, each in triplicates. *** *p* < 0.001.

We first analyzed the expression of β-amyloid (Aβ) peptide and Tau protein in PBMCs from mild AD, severe AD, and MCI patients compared to-matched control subjects (Figure 1). We observed similar levels of Aβ42 peptide in mild AD and severe AD cells which were three-fold higher compared to control samples (Figure 1A,B). The level of Aβ42 in PBMCs from MCI patients was similar to control subjects (Figure 1A,B). The expression of Tau protein was similar in the same cells obtained from mild AD and severe AD patients and two-fold higher compared to the level detected in the PBMCs of the control group (Figure 1A,C). No differences of Tau protein were observed in MCI patients compared to controls (Figure 1A,C).

The levels of Aβ peptide and Tau proteins were also investigated by the ELISA assay (Figure 1D,E). Again, we found comparable levels of expression of Aβ peptide in mild AD and severe AD (Figure 1D). In both groups Aβ peptide was up two/three-fold higher compared to control subjects and MCI patients (Figure 1D). No differences of Aβ peptide protein levels were observed in MCI patients compared to controls (Figure 1D). Comparable levels of the Tau protein were also confirmed in mild AD and severe AD (Figure 1E). Again, in both AD patients, Tau was up two/three-fold higher compared to control subjects and MCI patients (Figure 1E). No differences of Tau protein levels were observed in MCI patients compared to controls (Figure 1E).

### 2.3. Lysosomal Enzymes Levels Are Altered in PBMCs from Patients with Mild and Severe AD and MCI

**Figure 2:**
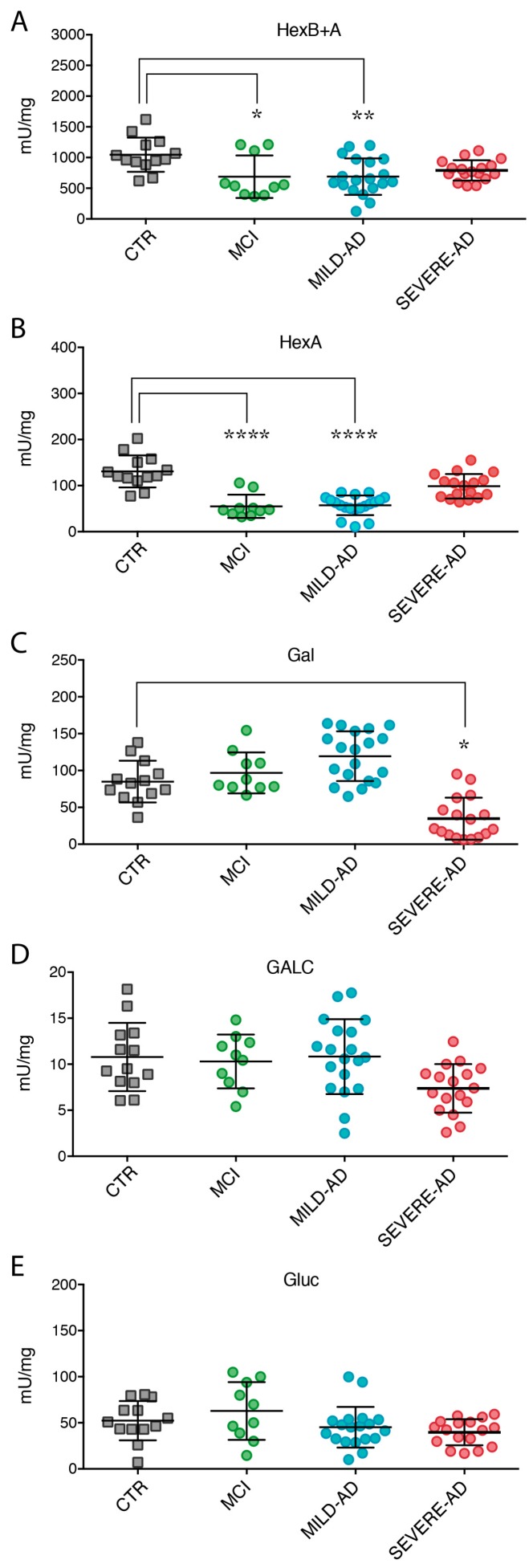
Lysosomal hydrolases activity (**A**–**E**) in PBMCs of mild AD, severe AD, MCI, and control subjects. Levels of hydrolases were measured by using specific fluorogenic substrates (see Section 4 for details). Results were expressed as the mean ± SEM of five independent experiments, each in triplicates. * *p* < 0.05, ** *p* < 0.01, **** *p* < 0.0001.

Hex activity was measured utilizing two different substrates: one hydrolyzed only by HexA and one hydrolyzed by both HexB and HexA isoenzymes [36], from now indicated as HexB + A activity. We found a significant reduction of Hex activity in the PBMCs of mild AD patients (HexB + A: 30% and HexA: 57%) when compared to the levels detected in the blood cells of control group (Figure 2A,B).

Noteworthy, a significant reduction of HexB + A (36%) and HexA (58%) activity in MCI patients was observed compared to control individuals. Yet, the difference between these groups was bigger when the HexA activity was measured (Figure 2A,B). Finally, HexA activity levels were reduced in MCI (19%) compared to severe AD patients (Figure 2A,B).

Intracellular levels of Gal activity were altered in AD patients. In particular, enzyme activity was significantly depleted in severe AD subjects (62% reduction) while minimally incremented in mild AD compared to the enzyme levels measured in the cells of control group (Figure 2C). A slight increased trend of Gal activity, comparable to levels measured in mild AD, was also detected in the samples of MCI subjects compared to control group (Figure 2C).

We showed a decreasing trend of GALC activity only in the PBMCs of severe AD subjects (Figure 2D). No differences were found in GALC activity in the mononuclear cells of MCI compared to control subjects (Figure 2D).

Finally, no Gluc activity changes were detected in all the samples of AD and MCI groups compared to control individuals (Figure 2E).

**Figure 3:**
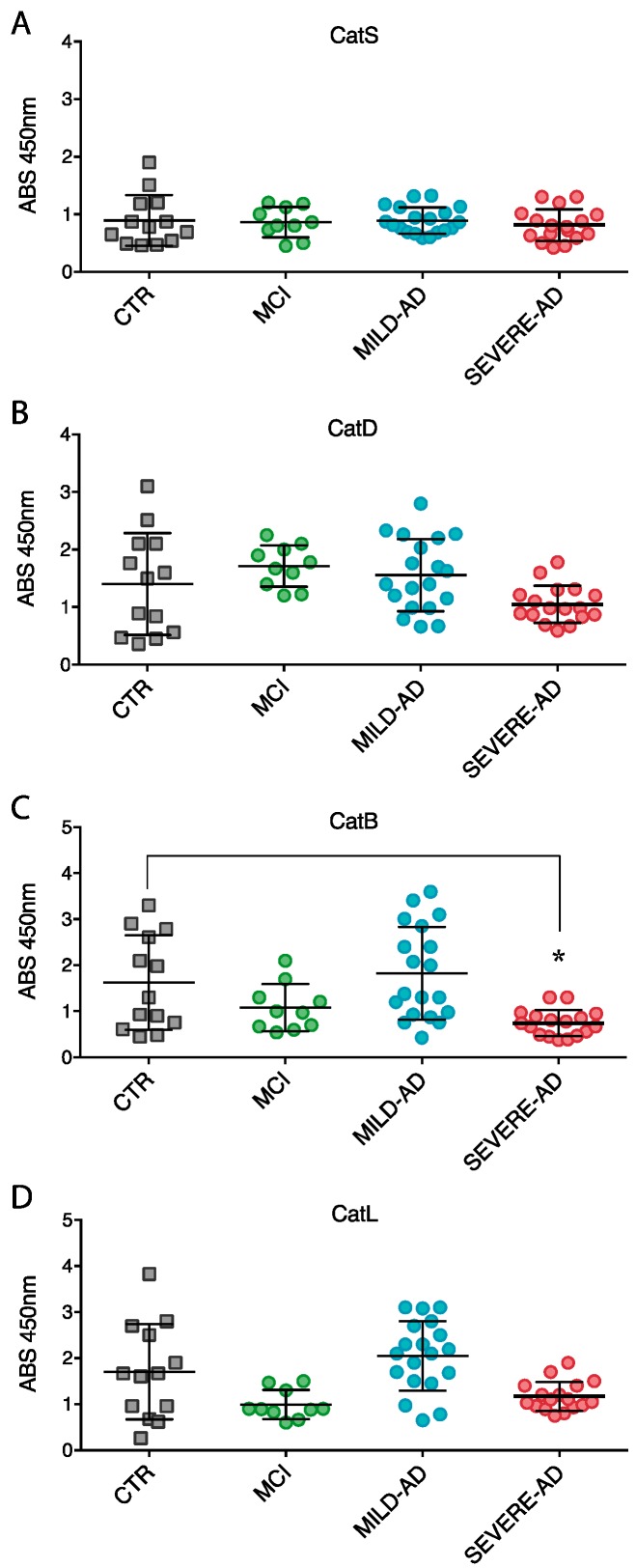
Lysosomal proteases expression (**A**–**D**) in PBMCs of mild AD, severe AD, MCI, and control subjects. Levels of proteases were determined by Western blotting. Shown are the densitometry measurements of all analyses. Results were expressed as the mean ± SEM of five independent experiments, each in triplicates. * *p* < 0.05.

The expression levels of cathepsins in PBMCs from all cohorts of subjects enrolled in the study were uniform (Figure 3). No statistical variation of CatS, CatD, and CatL expression levels were measured in MCI PBMCs with respect to control group (Figure 3A–D).

Only CatB expression in severe AD samples resulted significantly decreased (59%) compared to control and MCI groups (Figure 3C).

### 2.4. Secrete Lysosomal Enzymes Are Altered in Plasma from Patients with Mild and Severe AD and MCI

Characteristic of lysosomal enzymes is that at least 10% of their intracellular activity is secreted outside the cells through the secretory routes [36,46].

**Figure 4:**
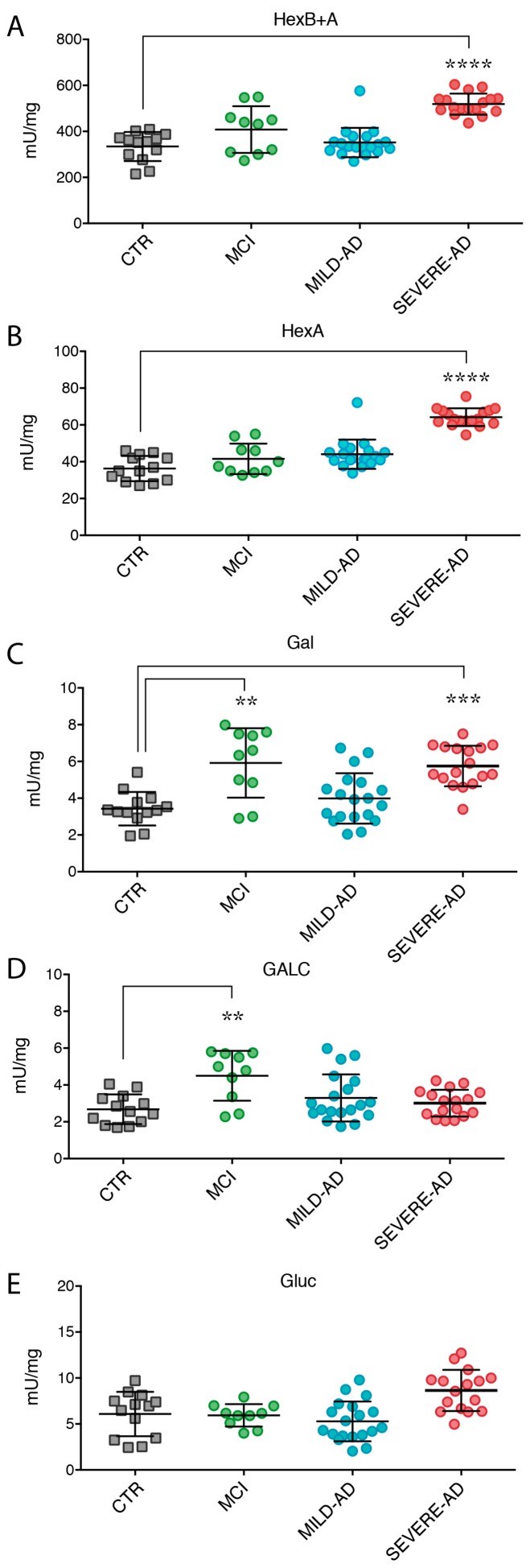
Lysosomal Hydrolases activity (**A**–**E**) in plasma of mild AD, severe AD, MCI, and control subjects. Levels of hydrolases were measured by using specific fluorogenic substrates (see Section 4 for details). Results were expressed as the mean ± SEM of five independent experiments, each in triplicates. ** *p* < 0.01, *** *p* < 0.001, **** *p* < 0.0001.

**Figure 5:**
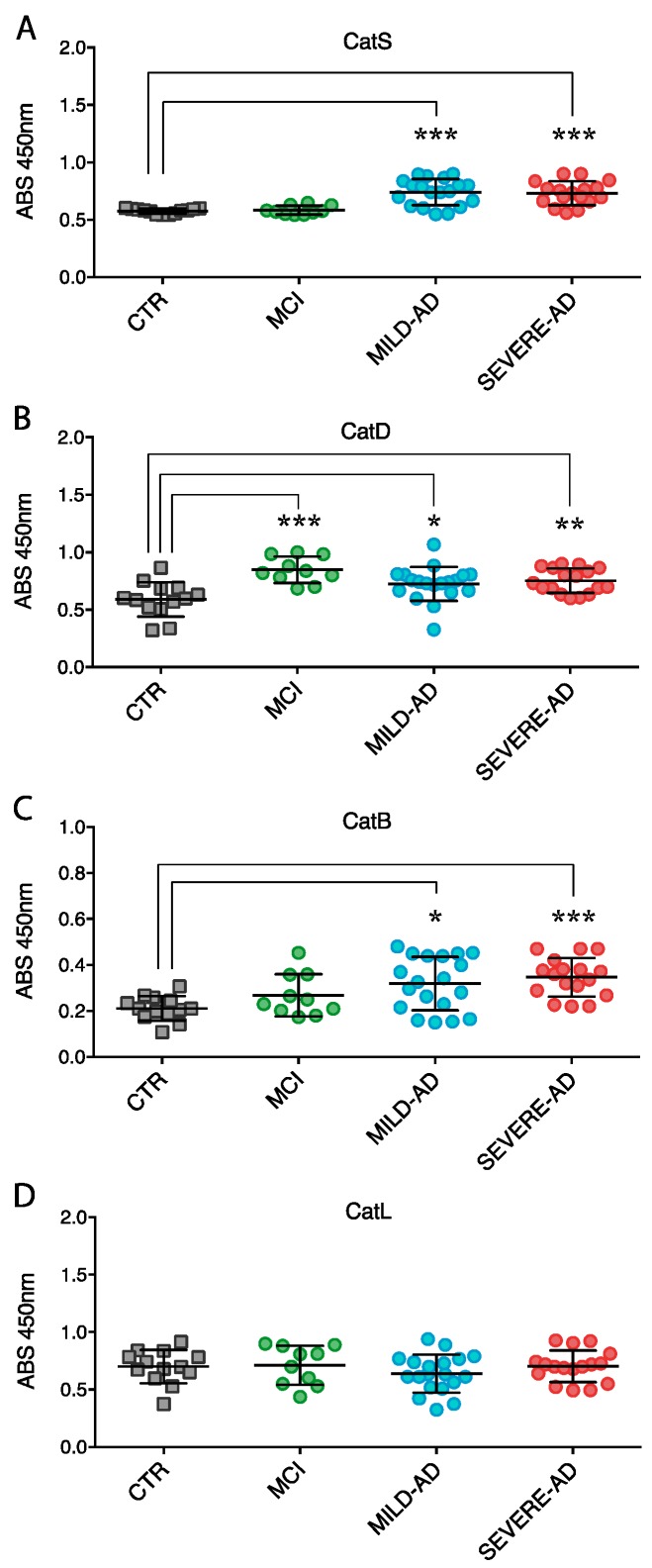
Lysosomal proteases expression (**A**–**D**) in plasma of mild AD, severe AD, MCI, and control subjects. Levels of proteases were determined in non-AD control group, mild AD and severe AD patients by using ELISA assay (see the Method section for details). Results were expressed as the mean ± SEM of five independent experiments, each in triplicates. * *p* < 0.05, ** *p* < 0.01, *** *p* < 0.001.

Therefore, we measured all glycohydrolases and proteases in blood plasma from mild AD, severe AD, MCI, and control subjects (Figure 4 and Figure 5).

A significant increase of secrete Hex activity (HexB + A: 52%, and HexA: 83%) was observed in plasma from severe AD patients compared to control subjects, while no variation was observed in mild AD (Figure 4A,B). Enzymes’ levels were also higher than those measured in MCI samples (Figure 4A,B). Moreover, an increasing trend of HexB + A enzyme activity was detected in the samples of MCI group compared to the control group (Figure 4A,B).

High levels of Gal activity were released in the plasma of severe AD (65% > CTR) and, in a lower extent, of mild AD patients (Figure 4C). Notably, a significant increase (71% > CTR) of secreted Gal activity was also detected in MCI patients (Figure 4C).

No secretion of GALC activity was detectable in the plasma of all AD groups (Figure 4D), whereas, an increase of GALC activity was measured in the plasma obtained from MCI patients (65% > CTR) (Figure 4D).

Finally, a minimal increase of Gluc activity was detected in samples of severe AD subjects compared to the values detected in the samples at milder stage of disease, control individuals, and MCI (Figure 4E).

The expression of secreted proteases was also measured in blood plasma from AD, MCI, and control subjects (Figure 5). To overcome the limitation of Wester Blotting procedures for evaluation of samples with highest protein content such as blood plasma, we developed in our laboratory an ELISA assay specific for proteases detection.

Secretion of CatS, CatD, and CatB proteins were significantly increased in mild AD (27%, 24%, 46%, respectively) and severe AD patients (23%, 28%, 69%, respectively) compared to control individuals (Figure 5A–C). Interestingly, AD levels of CatS and CatB were higher than levels found in the blood samples of MCI patients (Figure 5A,C). CatD levels were similar in AD and MCI patients and higher than control subjects (Figure 5B). Expression of CatL was almost unchanged (Figure 5B).

### 2.5. Comparison of Lysosomal Enzymes Expression Levels, Progression of AD and MCI

Finally, we collected the overall results from Figure 2, Figure 3, Figure 4 and Figure 5 and compared together the levels of lysosomal enzymes, stages of AD neurodegeneration and MCI status. We considered only enzyme levels that were significant different to control subjects (*p* < 0.05).

**Figure 6:**
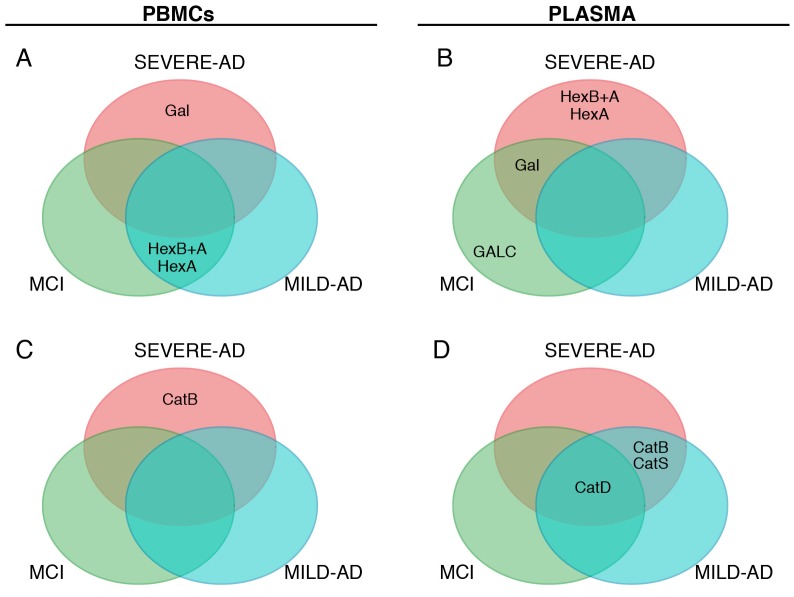
Lysosomal enzymes are able to discriminate mild AD, severe AD, MCI, and control subjects. The Venn diagram illustrated the relationship between mild AD, severe AD, and MCI groups, where lysosomal enzymes showed statistical variation with respect to control group (**A**–**D**).

A Venn diagram was used to illustrate our analysis. The diagram highlights that, among examined lysosomal enzymes, some specifically correlate with AD patients at different stages of progression of the disease, whereas others correlate with both AD group and others agree with MCI patients (Figure 6). Using PBMCs as the biological source, altered levels of Gal and CatB correlate with severe AD and not with mild AD or MCI patients (Figure 6A,C). Abnormal Hex (HexA+B and HexA) levels associated with mild AD and MCI and not with severe AD (Figure 6A). Using blood plasma as biological sample, Hex (HexA+B and HexA) alteration associated with severe AD and not with mild AD or MCI (Figure 6B). Abnormal Gal activity associated with severe AD and MCI and not with mild AD (Figure 6B). Changes of GALC activity correlated with MCI and not with severe AD or mild AD (Figure 6B), whereas those of CatB and CatS associated with AD patients and not with MCI (Figure 6D). Finally, CatD levels were altered in all AD and MCI groups (Figure 6D).

## 3. Discussion

This work demonstrates the altered expression of a several of lysosomal enzymes in peripheral blood samples of mild and severe AD compared to MCI patients and controls.

In the last two decades, association of lysosomal disturbances and neurodegeneration has been well documented [18,47,48,49]. Direct evidence came from the altered lysosomal glucocerebrosidase in Parkinson’s Disease (PD) patients. Classically, mutations in the glucocerebrosidase (*GBA1*) gene cause the Gaucher’s Disease, a genetic lysosomal disorder. However, recent findings indicated that at least 7–10% of PD patients might have a *GBA1* mutation and that mutations in this gene increased the risk above of 20- to 30-fold for the development of PD [48,50]. Additionally, the hallmarks of PD-*GBA1* disease are comparable to idiopathic PD [51].

Indirect evidence of lysosomal abnormalities and neurodegenerative diseases came from reports showing association of autophagy dysfunction and neurodegeneration. Physiologically, autophagy refers to a degradation machinery, consisting of a signal cascade of mechanisms taking play within the “lysosomal network”, and devote either to the control of removal of obsolete cellular constituents or macromolecule, or to the recycling of basic metabolites for new synthesis, or function as a “sensor” for the cellular health status. In neurodegenerative diseases, the autophagy pathway might be blocked at various steps along the signal cascade (selection, sequestration and lysosomal digestion of substrate) giving rise to distinct pathologic patterns that might have implications for the disease therapy [52]. For instance, autophagolysosomes impairment has been observed in fibroblasts of patients with Huntington’s disease [53], or in a murine cellular model of Amyotrophic Lateral Sclerosis [4,54] and in several models of AD [4,29,52,55].

Other indirect links between lysosomal aberrations and neurodegeneration came from data reporting the involvement of proteases in the proteolysis of Aβ-peptide [24,25] or showing the abnormal levels of Hexosaminidase and Galactosidases in cells from severe AD patients [22,26,27,30,31].

In this regard, our results confirm the lysosomal dysfunction in severe AD patients and add new findings on the association of abnormal levels of specific lysosomal enzymes with the mild AD and distinguish AD cases from MCI.

In PBMCs, Gal and CatB levels are associated with severe AD and not with mild AD and MCI patients, whereas Hex activity is associated with mild AD and MCI and not with severe AD. In plasma, Hex activity correlates with severe AD and not with mild AD and MCI; Gal activity associates with severe AD and MCI and not with mild AD; GALC activity agrees with MCI and not with mild and severe AD; CatB and CatS levels associate with both AD groups and not with MCI.

Altogether, our results compose a map where the combination of above enzymes levels in both PBMCs and plasma might help to recognize MCI from mild AD or both from severe AD and indicate that the difference although significant are not so clear to be used at the individual enzyme level (Figure 6). In fact, although activity levels of some enzymes (e.g., Hex, GAL) changed in mild and severe AD and MCI groups, the wide evaluation of both enzyme’s classes allows to identify the specific pathologic condition. The expression of altered levels of Hex in PBMCs without changes of GALC in plasma might indicate that those subjects are potentials mild AD. Abnormal plasma-levels of CatD without changes of plasma-CatB and -CatS might indicate that those cases are potentials MCI. Finally, altered levels of Hex in plasma and values of Gal and CatB in PBMCs might indicate that those subjects are potentially affected by severe AD.

Even if the alteration levels of above-mentioned lysosomal enzymes, to our knowledge, are secondary alteration of the diseases, it is likely that, in the peripheral blood (non-neurological biological samples), the lysosome compartment is more sensitive to AD cellular metabolic alteration when compared to the level of Aβ-peptide or Tau proteins (not modified in both AD groups analyzed). This observation supports the role of lysosomal enzymes as potential peripheral molecules that vary with the progression of AD [16]. Even if the definitive AD diagnosis must be confirmed with further neurologic examination (e.g., cognitive evaluation, neuropsychological assessments, etc.), the screening of new peripheral markers could improve the diagnosis, and reduce diagnostic time and health costs. This could be helpful for the identification of cases of AD at an early stage of the disease (mild AD) or MCI.

To our knowledge, our results are the first showing specific alteration of GALC in MCI patients. Absence of GALC activity causes the Krabbe Disease, a genetic lysosomal storage disorder affecting the white matter of the central and peripheral nervous systems as a consequence of the accumulation of un-degraded galactocerebroside [44,56]. We lack a clear explanation of the association of the increase of GALC activity in the plasma of MCI patients and more investigations are needed. However, we suggest that this event could correlate with the reduction of plasma ceramide (a product of hydrolysis from galactocerebroside and substrate of GalC), significantly reduced in MCI subjects [57,58].

We observed an opposite trend of the intracellular (reduction) and extracellular (increase) levels of the enzymes analyzed in AD and MCI patients compared to control subjects. We correlated these events as the response of lysosomes to the altered cellular microenvironment. An increase of the secretion rate of lysosomal enzymes is well-reported in several pathological conditions, such as neurodegenerative or inflammatory diseases [32,59,60]. Moreover, it is also described that PBMCs enhance the secretion of lysosomal enzymes under inflammatory or cytokine stimulation [58], like in AD [60,61]. Our data suggest a direct link between lysosomal enzymes alteration and AD as demonstrated by the non-homogenous scenario in terms of enzymes dysfunctions. In fact, whereas levels of Hex, Gal, GALC, CatB, CatD, and CatS changed in patients compared to healthy subjects, CatL and Glu content was unmodified in AD and MCI compared to control subjects.

Collectively, our results support the association of the lysosome dysfunction with neurodegenerative diseases and encourage studies aimed at exploring the molecular link between lysosomal enzymes and AD. In these regards, several research groups are exploring therapeutic interventions aiming at restoring lysosomal functions as useful treatment for AD and PD [52]. First evidence in murine models of AD suggests that the restoration of lysosomal proteolysis and the improvement of autophagy efficiency benefit neurological performances [62,63,64].

In conclusion, this study represents an easy and noninvasive method allowing measurement of a collection of lysosomal enzymes in a small peripheral blood sample from AD and MCI patients. The inclusion and exclusion criteria of the subjects’ enrollment make our findings robust and representative, notwithstanding the number of subjects included in the study. In fact, our system might function as a platform that might be expanded by enrolling more subjects and performing a wider expression analysis of more classes of lysosomal enzymes, including the autophagy’s enzymes. This might be helpful to identify new proteins having specific associations with the progression of AD or MCI or with other neurodegenerative diseases. In this regard, our results might have implications in developing regenerative medicine approaches for AD.

## 4. Materials and Methods

### 4.1. Biological Samples Collection

Blood human samples were taken after obtaining the informed consent from the patients or control subjects in accordance with the tenets of the Declaration of Helsinki, as part of a protocol approved by the Institutional Review Board of the Azienda Ospedaliera Universitaria Integrata N. 43320-04.09.2013 (Verona, Italy) [65].

The study included patients with MCI or AD recruited at the Neuromotor and Cognitive Rehabilitation Research Center (Department of Neurosciences, Biomedicine, and Movement Sciences), Azienda Ospedaliera Universitaria Integrata of Verona, and at the Mons. A. Mazzali Foundation, Mantua, Italy. Clinical diagnosis of MCI and AD was established according to the National Institute of Neurological and Communicative Disorders and Stroke, Alzheimer’s Disease, and Related Disorders Association (NINCDS-ADRDA) work group criteria for probable AD [66]. The severity of dementia was assessed by means of the Mini Mental Status Examination (MMSE) [67] and the Clinical Dementia Rating scale (CDR) [68,69]. The Neuropsychiatric Inventory (NPI) [70] was used to quantify the presence and severity of neuropsychiatric symptoms. Independence of the patients was evaluated with the level of instrumental activities of daily life (IADL) [71].

The AD patients were split in two groups based on the severity of dementia: patients with MMSE scores <15 and CDR 3 were classified as having severe AD (severe AD), those with MMSE scores between 15 and 24 and CDR 1–2 were classified as having mild AD (mild AD), while patients with MMSE scores >24 and CDR 0–0.5 were categorized as having MCI. As internal control of the study (CTR), a group of healthy individuals was recruited from the same geographical area. AD and CTR individuals were subjected to complete clinical and laboratory analyses. Exclusion criteria were: a history of depression or psychosis, alcohol or drug abuse, other neurological (e.g., Parkinson’s disease, multiple sclerosis, stroke brain injuries), orthopedic (e.g., osteoarthrosis), and cardiac or respiratory conditions (e.g., chronic obstructive pulmonary obstruction).

### 4.2. Sample Preparation

Venous peripheral blood (5 mL) was routinely collected between 9:00 a.m. and 10:00 a.m. from subjects in a fasting state and the samples were processed within 2 h to obtain plasma and peripheral blood mononuclear cells (PBMCs). Blood was drawn on ethylenediaminetetra-sacetic (EDTA) acid and diluted in phosphate-buffered saline (PBS) (Sigma-Aldrich, St. Louis, MO, USA) supplemented with 2 mM EDTA, pH 7.4 (PBS/EDTA), and layered on Lympholyte^®^ (Cedarlane Laboratories Limited, Burlington, ON, Canada) (density 1.077 g/mL) according to the manufacturer’s protocol. The interphase containing PBMCs was isolated and washed with PBS/EDTA at 300 g for 10 min at 21 °C. Plasma, representing the upper phase of the gradient, was collected.

### 4.3. Cell Extracts

Cells were washed in PBS, counted, re-suspended in 1 mL/10^6^ cells of ice-cold 10 mM sodium phosphate buffer, pH 6.0, containing 0.1% (*v*/*v*) Nonidet NP40 detergent, and further disrupted by sonication (three rounds of 30 s in ice cold tubes) using ultrasonic baths generating operating frequencies of 25–35 KHz. Procedures were carried out at 4 °C [25]. Proteins were measured by the Bradford method using bovine serum albumin as standard [35].

### 4.4. Enzyme Assays

Enzyme activities were measured using commercially available fluorimetric substrates. The hexosaminidase activity was determined with either 3 mM 4-methylumbelliferyl-*N*-acetyl-β-d-glucosaminide (MUG, Sigma-Aldrich, Milano, Italy) or 3 mM 4-methylumbelliferyl-6-sulfo-2-acetamido-2-deoxy-β-d-glucopyranoside (MUGS, Toronto Research Chemicals, Toronto, ON, Canada) substrates dissolved in 0.1 M-citrate/0.2 M disodium phosphate buffer, pH 4.5 [36]. The β-Galactosidase assay was performed using 1.5 mM 4-methylumbelliferyl-β-d-galactoside substrate (MUGAL, Sigma-Aldrich, Milano, Italy) resuspended in 0.1/0.2 M citrate/phosphate buffer, pH 4.0 [37]. β-Galactosylcerebrosidase assay was performed using 1.5 mM 4-methylumbelliferyl-β-d-galactoside substrate resuspended in 0.1/0.2 M citrate/phosphate buffer, pH 4.0 in the presence of 11 µM AgNO3 [38]. The β-Glucorinidase assay was performed using 1.5 mM 4-methylumbelliferyl-β-d-glucuronide (MUGlu, Sigma-Aldrich, Milano, Italy) substrate resuspended in 0.1/0.2 M citrate/phosphate buffer, pH 4.5 [37].

All reactions were performed using 50 µL of test sample mixed with 100 µL of substrate prior to incubation for 30 min at 37 °C. Ice-cold 0.2 M glycine/NaOH, pH 10.6, was used to stop the assays.

Liberated 4-methylumbelliferone was measured fluorometrically (λex 360 nm; λem 446 nm). One mU is defined as the amount of enzyme that hydrolyzes 1 nmol/min of substrate.

### 4.5. Western Blotting

Cell extracts containing 15 µg of proteins were separated by 12% SDS-PAGE and subjected to immunoblot as previously described [32]. The primary antibodies were: anti-App, -Tau, -Cathepsin S, -Cathepsin D, -Cathepsin B, -Cathepsin L antibodies (Santa Cruz, CA, USA). Immunostaining was performed using the ECL™ Detection System (GE Healthcare, Fairfield, CT, USA). Comparative analyses were carried-out by re-probing the same blot with different antibodies.

Densitometry analyses were performed by the Fiji software (Fiji Life-Line version, v.2015, National Institutes of Health, Bethesda, MD, USA). Relative band intensities were normalized to β-actin (Santa Cruz, CA, USA) as internal references. β-actin was also used to generate calibration curves for the corresponding quantitative tests [32]. Results are expressed as mean ± SEM of three independent experiments.

### 4.6. ELISA

Proteins (100 µg) were incubated in 96-well microplates (MaxiSorp^TM^, Nunc, Milano, Italy) in coating buffer (NaHCO_3_ 0.1 M pH 8.6) overnight at 4 °C. Unspecific binding sites were blocked with 300 µL of bovine serum albumin (3% diluted in Tris-buffered saline with 0.1% Tween-20) and incubated for 90 min at 37 °C. Following three washes with TBS + 0.05% Tween-20, the anti-Cathepsin S, -Cathepsin D, -Cathepsin B, -Cathepsin L, -App and -Tau antibodies (Santa Cruz, CA, USA.) were added to each well and the plate incubated overnight at 4 °C. After extensive washes with TBS + 0.05% Tween-20, each well was incubated with anti-human IgG -peroxidase-conjugated secondary antibody (Sigma-Aldrich, Milano, Italy) for 60 min at 37 °C. Immune-complex was measured using the peroxidase substrate T0440 3,3′,5,5′-tetramethylbenzidine (Sigma-Aldrich) and the optical density was measured at 450 nm using a microplate reader (DV-990BV6; GDV, Rome, Italy). All assays were tested in triplicate. Statistical analysis was carried out using GraphPad 4.0 Software (San Diego, CA, USA).

### 4.7. Statistical Analysis

Results are expressed as means ± SEM. Based on non-parametric distributions of values, data analysis was carried out using the Kruskal-Wallis nonparametric one-way ANOVA that does not require the fulfilment of assumptions of normal distribution, interval data and homogeneity of group variance. A pairwise comparison test was carried out using Dunn-Bonferroni’s Multiple Comparison Test for any dependent variables for which the Kruskal-Wallis test was significant (GraphPad 6.0 Software, San Diego, CA, USA). A * *p* < 0.05 was considered significant.

## Figures and Tables

**Table 1:** Clinical characteristics of the study participants.

PARAMETERS	CTR	MCI	MILD AD	SEVERE-AD
Weight (kg)	73 ± 12	75 ± 19	73 ± 12	73 ± 13
Height (m)	1.67 ± 0.1	1.65 ± 0.1	1.58 ± 0.1	1.62 ± 0.1
SBP (mm Hg)	129 ± 33	136 ± 37	132 ± 12	130 ± 9
DBP (mm Hg)	90 ± 24	92 ± 12	86 ± 10	90 ± 5
Glucose (mg∙dL^−1^)	95 ± 32	107 ± 21 †	91 ± 8	95 ± 47
HDL (mg∙dL^−1^)	50 ± 17	58 ± 19	57 ± 21	57 ± 11
LDL (mg∙dL^−1^)	100 ± 23	102 ± 15	122 ± 32 †	110 ± 12
Pharmacological treatment *n*. (%)				
Cholinesterase Inhibitors	0	2 (10) †	10 (42) †	5 (25) †
Antipsychotics	0	0	1 (4)	1 (5)
Antidepressants	0	0	2 (8)	4 (20) †
Benzodiazepines	0	0	0	1 (5)
Comorbidity *n*. (%)				
CVD	0	1 (5)	4 (16) †	2 (10) †
Diabetes	0	1 (5)	0	1 (5)

Mild Cognitive Impairment; AD, Alzheimer’s Disease; SBP, systolic blood pressure; DBP, diastolic blood pressure; HDL, high-density lipoprotein; LDL low-density lipoprotein; CVD, cardiovascular diseases. Values are expressed as mean ± standard deviation (or percentage in brackets). † *p* < 0.05 vs. CTR.
